# Current Trends in the Evaluation of Osteochondral Lesion Treatments: Histology, Histomorphometry, and Biomechanics in Preclinical Models

**DOI:** 10.1155/2019/4040236

**Published:** 2019-10-09

**Authors:** M. Maglio, S. Brogini, S. Pagani, G. Giavaresi, M. Tschon

**Affiliations:** IRCCS-Istituto Ortopedico Rizzoli, Laboratory of Preclinical and Surgical Studies, via di Barbiano 1/10, 40136 Bologna, Italy

## Abstract

Osteochondral lesions (OCs) are typically of traumatic origins but are also caused by degenerative conditions, in primis osteoarthritis (OA). On the other side, OC lesions themselves, getting worse over time, can lead to OA, indicating that chondral and OC defects represent a risk factor for the onset of the pathology. Many animal models have been set up for years for the study of OC regeneration, being successfully employed to test different treatment strategies, from biomaterials and cells to physical and biological adjuvant therapies. These studies rely on a plethora of post-explant investigations ranging from histological and histomorphometric analyses to biomechanical ones. The present review aims to analyze the methods employed for the evaluation of OC treatments in each animal model by screening literature data within the last 10 years. According to the selected research criteria performed in two databases, 60 works were included. Data revealed that lapine (50% of studies) and ovine (23% of studies) models are predominant, and knee joints are the most used anatomical locations for creating OC defects. Analyses are mostly conducted on paraffin-embedded samples in order to perform histological/histomorphometric analyses by applying semiquantitative scoring systems and on fresh samples in order to perform biomechanical investigations by indentation tests on articular cartilage. Instead, a great heterogeneity is pointed out in terms of OC defect dimensions and animal's age. The choice of experimental times is generally adequate for the animal models adopted, although few studies adopt very long experimental times. Improvements in data reporting and in standardization of protocols would be desirable for a better comparison of results and for ethical reasons related to appropriate and successful animal experimentation.

## 1. Introduction

The treatment of osteochondral (OC) defects is still a great challenge in the orthopaedic field. Whatever the triggering cause of OC lesion formation is (osteonecrosis, osteoarthritis, sports-related injuries, and chronic overload), the progression of the lesion leads to the destruction of the normal architecture of the affected district, both in the cartilaginous component and in the subchondral bone, further aggravating the pathological picture of osteoarthritis, if already present or promoting its onset. Consequently, the functionality of the affected joint is compromised by mechanical and tribological alterations which, in the final stage, can require invasive surgical approach up to total joint replacement [1].

It is difficult to give precise numbers about the incidence of OC lesions; however, it has been observed that in about 60% of patients undergoing various types of knee surgery, it is possible to find OC lesions, frequently in the medial femoral condyle and usually involving subchondral bone [2]. Moreover, these lesions also characterize the idiopathic process of osteochondritis dissecans, which can occur from childhood through adult life in approximately 15 to 29 per 100,000 patients and for which a number of possible causes has been reported including repetitive microtrauma, vascular abnormalities, and genetic predisposition [3, 4]. The deep and well-known structural, biochemical, and biomechanical differences between cartilage and subchondral bone have prompted much of the past research in the regeneration of each compartment separately. However, cartilage and subchondral bones are biologically and functionally linked, influencing each other physiologically and pathophysiologically to form what is considered the OC unit [5].

The observation of the OC unit detects a complex system, with great variations in terms of functions and architecture, with a progressive heterogeneity of cellular component, aggrecan presence, collagen types and contents, and cartilage fibrils thickness, starting from the articular surface up to the bone. The biomechanical skills of the districts are closely related to the mutual interaction between the various parts of the OC unit. Articular cartilage is mainly responsible for absorbing impacts, but the ability to manage weight bearing and load is strengthened by subchondral bone, also because of its key role in providing nutrition to cartilage [6].

Consequently, the complexity of the OC unit makes the approach to the setup of regenerative medicine studies quite demanding. The use of multilayered and bi/triphasic scaffolds tries to address the need to restore the functionality of this district, considering both bony and cartilaginous features and the strict dependence between chondral and subchondral status. In the last years, the combination of such scaffolds with cells from different sources seemed to be a promising approach, exploiting the ability of stem cells to differentiate towards different lineages without immunogenic effects [7].

A wide variety of animal models are employed in this research field, and the most common anatomical site in which OC lesions are created is the stifle joints (both medial and lateral condyles and trochlea). The correct dimensions of such defects, in order to obtain a lesion which cannot spontaneously heal but which, at the same time, is not so wide as to affect the effectiveness of the treatment, are still a topic of discussion. In the literature, for each animal model, lesions of very different dimensions are found, involving or not the subchondral bone. Such a variety of in vivo protocols makes it difficult to establish a standard model as well as to compare results from different studies, also because the posttreatment evaluations can vary a lot among studies [8]. The complexity of the OC district gives the possibility of using a large number of assessments, ranging from histological stains specific for bone and cartilage to specific markers for cartilage regeneration/degeneration, new bone formation, mineralization status etc. As for any regenerative medicine study, even those related to OC regeneration may be enriched by biomechanical assessments. Although these are generally destructive tests and therefore require a greater number of animals if they are to be combined with histological evaluations, their use is fundamental for an assessment of the quality of the regenerated tissue. It appears particularly important considering that OC lesions are generally located in joints subjected to mechanical loading, so that the resumption of a correct mechanical competence is essential to define the success of a treatment [9].

To have an overview about the current trend for the evaluation of treatments for OC regeneration, the recent literature about in vivo models of OC defects were reviewed, focussing on the assessments performed in terms of histological, histomorphometrical, and biomechanical evaluations.

## 2. Methods

### 2.1. Descriptive Systematic Literature Review

This systematic review was carried out according to PRISMA guidelines. Electronic database searches were performed on http://www.pubmed.com and http://www.webofknowledge.com to identify studies reporting the following key terms: (osteochondral scaffold OR osteochondral biomaterial OR osteochondral regeneration OR osteochondral tissue engineering OR osteochondral defect OR osteochondral lesion) AND (biomechanics OR biomechanical evaluation OR biomechanical test OR histomorphometric evaluation OR histomorphometric analysis OR histomorphometric test OR histomorphometry). Study eligibility was independently determined by reviewing titles and abstracts using the following inclusion criteria: preclinical studies of any level of evidence, full text, English language, and reports published from April 2009 to April 2019. Exclusion criteria were articles not in English, reviews, not in vivo studies, papers not reporting histological, histomorphometric, or biomechanical assessments, papers involving only chondral lesions, and duplicate papers. Three independent researchers performed both the screening step and subsequent data extraction (MM, SB, and MT).

### 2.2. Data Extraction and Management

From all studies, specific data related to the adopted experimental animal model (type and number of animals), experimental setup (site of implant, OC lesion dimension, and experimental time), type of treatment, performed histological/histomorphometric and biomechanical evaluations, and main results were extracted (Tables 1 and 2).

## 3. Results

### 3.1. Literature Results

**Figure 1:**
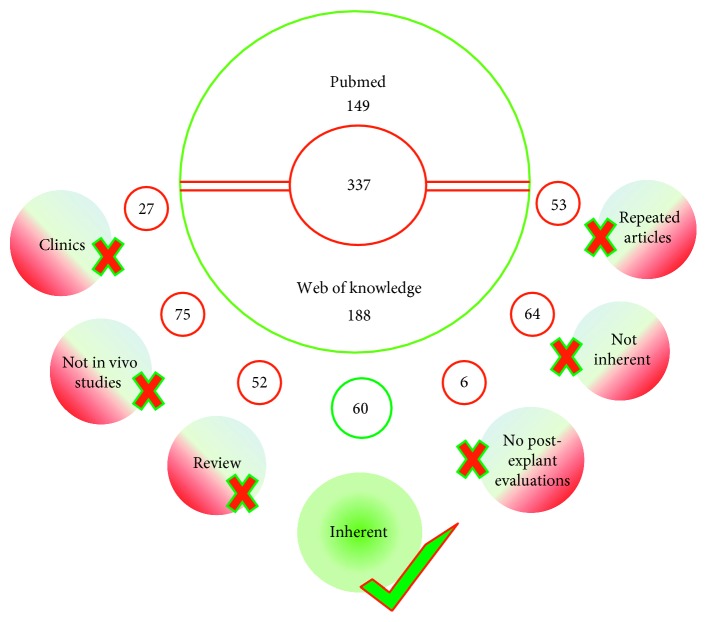
Flowchart of research strategy and paper selection.

The a priori search retrieved 149 articles from http://www.pubmed.com and 188 from http://www.webofknowledge.com. After screening, several articles (224) were excluded: 27 were clinical studies, 75 were not in vivo (in vitro and ex vivo studies, cadaveric studies), 52 were reviews, 6 did not report histological, histomorphometric, and biomechanical evaluations, and 64 were not related to the research (chondral only implants, ectopic implants, and mathematical models). Therefore, a total of 113 papers were recognized eligible for the review and after the use of a public reference manager (Mendeley 1.19.3) to eliminate duplicate articles; 60 papers remained: 33 performed in small-medium animal models (rodent and lapine) and 27 in large animal models (canine, swine, equine, and ovine) (Figure 1).

### 3.2. Rodent Model

Among the retrieved papers, three evaluated the osteochondral tissue regeneration of the joint by adopting a rat animal model. All papers selected the same anatomical site of implant in the trochlear groove, had similar follow up times of 1-2 months, performed defects with similar dimensions (diameter range 1.4–2 mm and depth range 1–1.5 mm), and conducted histological/histomorphometric analyses on paraffin-embedded samples. The study by Zhang et al. was the only one that conducted both histological/histomorphometric and biomechanical analyses [10]. These analyses found similar significant results between the histomorphometric score (ICRS score) and biomechanical analysis of Young's modulus of the regenerated cartilage. Investigations on biomechanics were performed by the indentation test on freshly excised samples submerged in PBS without any inclusion, whereas the histological scoring system was made on decalcified paraffin-embedded samples.

Instead, the other two works by Mendes et al. and Lin et al. in a low weight-bearing area of the joint performed microtomographic analysis and histological score (Wakitani score), respectively, but not biomechanical evaluations of the regenerated tissue. So far, a direct comparison of results is not possible [11, 12].

### 3.3. Lapine Model

Thirty studies of the retried papers involved rabbits as an animal model [13–42]. The New Zealand white rabbit was adopted in 28/30 studies, whereas Japanese rabbit was used in two related studies [38, 39]. The majority of the researches claimed to use skeletally mature animals (range age: 6–32 months). However, in some studies [14, 24, 36, 37], a lower animal age until 3 months was reported. Of note, an average weight between 2.5 and 4 kg was reported in all these studies except for Cheuk et al. [24]. Finally, regarding the animal age, it is to stress that Martin-Hernandez et al. openly declared the use of 3-month-old skeletally immature rabbits [30].

Medial and/or lateral femoral condyles (14/30) and the trochlear groove (16/30) have been the selected anatomical sites where osteochondral defects were created. Between the defects created in the femoral condyles, 3 were made in the load bearing areas [13, 24, 25]. The defects dimension varied from 1.5 mm to 6 mm in diameter and from 1.5 to 10 mm in depth contributing to make the comparison difficult. Notably, the fact that, in some studies, the surgical defect performed was defined as a “full thickness” cartilage defect, but the range (diameter: 3–5 mm; depth: 2–10 mm), both in diameter and in depth of the defect, varied among studies [9, 20, 30, 31, 34, 37], indicating that an accordance is not reached yet on the issue. In terms of defect size, an extremely variation was noticed also in the experimental times, ranging from 1 day [23] to 36 weeks [18]. In 12/60 papers, the chosen experimental time was 1–1.5 months, but in the 75% of these works, also longer experimental times were taken. Overall, the most common long-term experimental times were 3 (8/30) and 6 months (7/30 papers) and only one paper arrived up to 9 months.

Except for two studies [31, 39] where only biomechanical tests were performed, all authors (28/30) conducted histological/histomorphometric analyses mainly on decalcified and paraffin-embedded (25 papers out of 30) samples. In two studies, such an analysis was performed on frozen samples [22, 23], while in three studies, 2-hydroxyethyl methacrylate (Technovit) and polymethylmetacrilate were chosen as embedded solution, respectively [27, 29, 40]. Apart from the research of Wada et al., both embedding methods were adopted [38]. Mainly, histologic analysis included the adoption of semiquantitative scoring systems both for macro- and microevaluation of samples. Where specified, both in gross evaluation and microscopic analysis, different scoring systems were adopted. Between them, the O'Driscoll score resulted as the most utilized (12 to 30), immediately followed by the ICRS (5 papers out of 30) and Niederauer (3/30) scores. As basic histological staining for the general assessment of cell and tissue morphology, the common hematoxylin-eosin (H&E) staining was used. Instead, both Safranin O (associated or not with the fast green dye for the bone compartment) and Toluidine Blue were the staining methods most frequently chosen for proteoglycans as well as glycosaminoglycans content, in addition to the Alcian Blue staining method. Only five studies utilized the two classical techniques to visualize collagen fibers in histological section, picrosrius red [35] or Goldner's trichrome [16, 18, 21, 30]. Finally, for undecalcified samples, the Stevenel Blue/Van Gieson Pichrofucsin dye [29], thionine staining [27], and Toluidine Blue were used to evaluate the osteochondral compartment. Where specified, all the abovementioned histological stainings were performed after cutting the samples according to a sagittal [14–18, 22–25, 27–30, 38] or longitudinal [13, 40, 41] plane.

Histomorphometry mainly included the quantification of osteochondral repair tissue, both of cartilage and bone compartments, microtomographic bone-related parameters, and the percentage of biochemical analytes such as collagen type I and II or proteoglycan content. Different from commonly performed analyses, one study reported osteoclasts quantification also after TRAP staining [23]; other two researches quantified cartilage specific gene expression and sulphated glycosaminoglycan (sGAG) [33, 37] or DNA content [37], as well as protein expression by mean western blot in the in vivo constructs [41]. Finally, only one study performed quantification of oxytetracycline incorporation [29]. Between imaging techniques, MRI [35] and SEM [40] was also adopted together with microCT.

Biomechanical evaluations on fresh samples or previously frozen samples [31, 32] implemented the histomorphometric results of the abovementioned studies.

Between biomechanics methods, indentation tests were used to evaluate parameters such as cartilage stiffness, instant modulus and shear modulus, compressive strength, Young's modulus, or contact stress of the cartilage compartment. Among the studies where biomechanics was performed, three studies specified which type between nanoindentation [35] or microindentation [20, 34] was used. Finally, the pushout test was conducted in two related studies [38, 39].

As far as biomechanical results were concerned, Schmal et al., Betz et al., and Zhang et al. found similar trend between histological and mechanical analysis [15, 19, 26]. In the work of Schmal et al., cartilage thickness, instant modulus, and shear modulus on fresh samples by mean indentation tests were evaluated while histomorphometry was conducted by quantification of collagen content and by adopting the semiquantitative OARSI score [15]. Mechanical stiffness and quantification of bone area within the subchondral defect and the O'Driscoll score were instead evaluated by Betz et al., finding a positive correlation between the histomorphometric parameters and higher stiffness values [19]. Significant differences between the experimental groups in comparison to other groups were found in biomechanical and microCT evaluations by Zhang et al. in [26]. Cao et al. assessed a significant higher chondrocyte viability, PG content, type II collagen, and Young's modulus on trochlea defects treated with osteochondral allografts stored in a particular medium. However, no significant results were detected in comparison to fresh osteochondral allografts [42].

On the contrary, the work by Kazemnejad et al. did not find any significant differences in quantitative histopathological and mechanical data [18].

### 3.4. Canine Model

Six papers analyzed the osteochondral regeneration of defects made in the knee of dogs [43–48]. Different from the studies conducted in rats, the most (5 out of 6) performed defects in the high load bearing areas in medial or lateral femoral condyles and one in the low load bearing area in the patellar groove. The dog's age was quite homogeneous (range 1.5–5 years), with the exception of two studies where dog ages were not reported [43, 44]. On the contrary, defect dimensions greatly varied from 3.5 to 11 mm in diameter and from 2 to 10 mm in depth. Also by analysing the defect dimensions in relation to the animal's breed, the three papers that used Mongrel dogs performed defects in femoral condyles with diameters ranging from 3.5 to 8 mm and height from 8 to 10 mm [43, 47, 48].

The experimental times ranged from 3 to 12 months; however, most of the papers (4 out of 6) selected the six-month period for investigations. All authors performed both histological/histomorphometric and biomechanical tests: histology was performed on decalcified and paraffin-embedded samples and biomechanics on fresh or frozen/thawed samples (4/6 papers among which 3 submerged samples in saline) or frozen/thawed samples (2/6 papers). Mainly, histology included the adoption of semiquantitative scoring systems, histomorphometry measured microtomographic bone-related parameters and quantification of biochemical analytes (collagen and/or GAGs), and biomechanics evaluated cartilage stiffness or modulus by performing the microindentation test. One work by McCarty et al. included also a MRI evaluation of MOCART score and the quantitative T2 mapping [44]. A great concern about the examined studies is that the study reporting is almost incomplete, because in some cases, there is a lack of indications about the animal's breed, age, or anatomical plane of cut.

As far as results were concerned, Quiang et al. [43] and Baba et al. [45] found similar trends between histological and biomechanical analyses; both works measured the cartilage stiffness by means of the indentation test, whereas in the work of Quiang, histomorphometry was conducted by measuring the 3D bone volume by microCT, and in the work of Baba, semiquantitative Niederauer and Changoor scores were adopted.

Yang et al. and Cook et al. found significant results only in histological/histomorphometric investigations and not in biomechanics, whereas the remaining two papers did not find any statistical significance in any of the performed analyses [46, 47].

### 3.5. Swine Model

Six papers [49–54] employed a swine animal model to investigate the repair and regeneration of the joint tissues: two works were made in 6-month-old pigs [49, 50] and four in miniaturized pigs with ages ranging from 7 months to 2.5 years [51–54]. The experimental times ranged from 1.5 to 13 months, but four out of six papers selected the 6-month endpoint.

In pigs, critical sized osteochondral defects, meaning that they were not able to spontaneously heal, were created. These studies found similar significant trend between histological and histomorphometric and biomechanical investigations that were conducted on paraffin-embedded samples (longitudinally cut) by adopting semiquantitative scoring systems and by the indentation test to evaluate Young's modulus. Interestingly, Ho et al. performed defects both in the high-load bearing femoral condyles and in the low-load bearing trochlear groove, finding that, for the same treatment, mechanical stimulus had beneficial effects on the tissue regeneration gaining superior healing in the condyles than in the trochlea [50]. Moreover, Ho et al. correlated the obtained results from the histomorphometric score and biomechanical test: they found positive correlation between the bone regeneration measured by microCT and Young's modulus measured by the indentation test at the two different implantation sites [50]. This study suggests a close interaction in the healing of both tissues as cartilage repair is dependent on the underlying bone for support and both histomorphometric and biomechanical tests are able to identify such improvements.

Among the four works in minipigs, three used Goettinger minipigs: all defects were created in the trochlear groove with dimension ranging from 5.4 to 7 mm in diameter and from 8 to 10 mm in depth [51–54]. Christensen et al. and Jung et al. performed histological/histomorphometric analysis on resin-embedded [51] and paraffin-embedded samples [52], respectively, but not biomechanical ones. Gotterbarm et al. and Zuo et al. quantitatively measured the osteochondral regeneration by histological scores on paraffin-embedded samples and the compressive modulus of regenerated cartilage in fresh samples by the indentation test [53, 54]. While Gotterbarm et al. failed to observe significant differences in any of the measured parameters, Zuo et al. found corresponding trends between biomechanics and histomorphometry [53, 54].

### 3.6. Equine Model

One work analyzed the knee tissue regeneration in an equine animal model: osteochondral defects with a smaller and deeper core was adopted in adult ponies for the localized delivery of gene transfer vectors [55]. The work dealt with paraffin-embedded histological samples, and the follow-up experimental times were 3 and 6 months for MRI and CT investigations and 13 months for microCT and histological ones. However, the authors did not perform biomechanical analysis, and they found correspondence between MRI and clinical CT data [55]. The paucity of data regarding the use of this model, owing to huge costs and ethical reasons as companion animals, affects the relevance of their results.

### 3.7. Ovine Model

Among papers retrieved after research, 14 resulted to involve ovine models [29, 56–68]. Five papers out of 14 used goats, while 9 used sheep. Among works dealing with goats, 2 used Dutch milk goats while the remaining three did not report the breed. Among works dealing with sheep, 9 used Merino, 1 Sardinian, and 1 Arcott and 2 papers did not report the breed.

In contrast with an almost generalized use of macroscopic and microscopic scores (mainly ICRS score and O'Driscoll score) and histological analyses, in 7/14 papers, microtomographical assessment is reported, and in 5/14 papers, biomechanical tests are performed; among these, 4/5 were indentation tests and 1/5 compression test. When specified, the site selected for the creation of osteochondral defects was in one case the talus [56], in one paper, the trochlea and the medial condyles [57], and for the other papers, medial condyles [58–62] or both medial and lateral condyles [29, 63–75]. In some cases, the choice of central weight-bearing area was underlined [60, 61, 66, 67].

It is noticeable the wide range of defect dimensions, from 2 mm to 9.4 mm of diameter and from 11 mm to 25 mm of depth, which make it difficult to compare the studies with each other. By analysing the defect size in relation to the species, the same variability exists. In the femoral condyles of goats, the dimensions were quite homogeneous (diameter range was 5–6 mm and the height range 3.5–8 mm), while in the femoral condyles of sheep, the diameter range was 2–9.4 mm and the height range was 2–25 mm. By grouping data per breed, defects in the femoral condyles of Merino sheep (the mostly used breed in 5/9 studies of sheep) had a diameter range of 4–9.4 mm and an height range of 11–25 mm, suggesting there is no relation to the breed.

With the exception of the paper by Bell et al. in which very short experimental times were selected (one day and 3 weeks) [61], for the other studies, a minimum of 1 month to a maximum of 24 months of experimental times were chosen, but most of the studies (11/14) selected 6 months. In almost all papers (11/14), immunohistochemical evaluations were performed to investigate mainly collagen I and II reactivities, while the paper by van Bergen is the only one in which evaluation of bone mineralization was performed with MAR measure [56]. In a single case, the possibility to extract RNA from paraffin-embedded samples is exploited to perform gene expression analyses [67].

In all papers, biomechanical tests were performed before histological analyses on fresh samples, except for the paper by Mayr et al. in which PMMA embedding was performed, and achieved load, absorbed energy, and contact stiffness were evaluated [68]. Jia et al. [62] and Jurgens et al. [57] measured Young's modulus, keeping samples in PBS at room temperature during the compression and indentation tests. Marquass et al. also tried to mimic a physiological environment during the test using a polymethylmethacrilate (PMMA) tank filled with PBS (7.4 pH) [59]. Articular cartilage deformation was tested by Manunta et al. [65].

As for the region of interest evaluated, when clearly specified, Mayr et al. performed measurements both in the implant and healthy tissue at the interface with the defect [68], while Marquass et al. considered an area next to the centre of the defect [59]. As control, Marquass et al. compared the results with those obtained from tests previously performed on untreated joints, while Jurgens et al. tested the native cartilage adjacent to the osteochondral defect [57]. One paper [68] compared results with those obtained from the contralateral untreated defect.

Generally speaking, except for the cases in which the biomechanical test did not show significant differences among groups [56, 57, 59], the results from mechanical assessments are comparable with those obtained from histological evaluations.

## 4. Discussion

The progress of histological techniques and technological advances related to image analysis software and specific test equipment has allowed the study of the musculoskeletal tissue and the evaluation of regenerative medicine protocols more thorough and complete. The peculiarity of the OC unit, due to the presence of both cartilaginous and bone tissues, makes its evaluation quite complex and varied [69, 70]. One of the most challenging aspects can be considered the biomechanical evaluation as the different structural characteristics of bone and cartilage, even if only considering the difference in extracellular matrix composition. It requires the setup of protocols being able to perform a correct measure in relationship to the tissue and the loading expected. Consequently, it is not so rare that post-explant assessments focus mainly on one tissue rather than another, although it is now established that a full-fledged consideration of the district is indispensable for the evaluation of a new therapy or treatment [71].

As confirmed by data extracted from this review, paraffin embedding still remains the most common embedding technique for histological evaluation. The types of treatment tested in the reviewed papers allow an easy application of this technique. In fact, the majority of the studies tested the effect of cells, mainly mesenchymal stem cells from different sources, and also chondroblasts or chondrocytes. When used in combination with scaffolds/biomaterials, the choice frequently fell on collagenic/gelatin scaffolds, biphasic constructs, and rarely ceramic or PLGA materials. Most of these materials can be easily embedded in paraffin, which has also the advantage to allow a better evaluation of cellularity and cartilage status, allowing to perform specific stainings and also immunohistochemistry evaluations. Resin embedding was applied mainly in the few cases in which hard materials, such as metals, were used. The paucity of works using resin embedding protocols might be due to the need of specific costly equipment, in particular for cutting samples, such as rotating microtomes and oscillating saws. In addition, although resin inclusion allows the study of the material-bone interface without decalcification and maintaining any type of implant in place, it is less suitable for analysing bone cellularity and cell morphology, with a different yield of histological stainings. Among these, hematoxylin/eosin and Safranin O/fast green stainings are the most adopted techniques for the OC tissue, regardless of the animal model employed. The first one is probably one of the most commonly used histological stainings. The chromatic gradation that the staining can take according to the degree of tissues mineralization makes it particularly suitable for the study of the OC district, highlighting both the presence of calcification and the predominantly collagenic areas. On the other hand, the specificity and stoichiometric affinity of Safranin O for proteoglycans make this dye ideal for the evaluation of cartilage status and for the application of most of the scores for OC regeneration [72]. There are many existing scores for the evaluation of OC regeneration, and the data extracted from the review show that these are all widely used, from the simplest and with less parameters considered (e.g., Pineda and Wakitani scores) to the more complex and complete ones (e.g., O'Driscoll, Sellers, and Forties scores). ICRS and OARSI scores are quite commonly used, sometimes in combination, although the first is usually applied for the evaluation of human joints, while the second is more specific for the evaluation of osteoarthritic cartilage staging [73]. The existing scores for OC evaluation share some fundamental parameters, for example, cell morphology, filling of the defect, and staining of matrix. Both O'Driscoll and Sellers scores specify that the extent of matrix staining is expected to be evaluated with Safranin O staining, while in the Fortier score, Toludine Blue staining is suggested for the evaluation of adjacent cartilage. This last parameter is actually evaluated only in two scores (Wakitani and Fortier), while the evaluation of subchondral bone is provided only by Sellers score. This evidence underlines a relevant gap in the perspective of a comprehensive evaluation of OC unit regeneration and suggests the need to employ at the same time different scores to evaluate all critical aspects. However, the choice to perform more than a single score seems to be not frequently adopted, while quantitative evaluations as microCT or histomorphometric measures or immunohistochemical staining are frequently used, in addition to histological scores. For an exhaustive evaluation of cartilage regeneration and acquisition of hyaline characteristics, the peculiar composition of articular cartilage requires the evaluation of both collagen I and II presence. To make the picture complete, collagen X quantification provides information about the process of cartilage calcification and bone growth, thus allowing comprehensive evaluation of both cartilagineous and bone tissues [74]. Due to the emerging role of the subchondral bone, microCT analyses have been widely performed for the evaluation of bone volume, microarchitecture, and response to treatments.

An interesting aspect emerged by the results is the widespread use of biomechanical tests in the evaluation of OC regeneration although mostly performed on the superficial articular cartilage [75]. Biomechanical tests require special equipment and are usually associated with sample destruction or alteration, unlike microtomographic assessment, which has benefits to be a nondestructive technique, so that fewer studies could have been expected involving this kind of evaluation. Considering that hard resin embedding allows to perform some mechanical evaluations, as microhardness and indentation tests, it could be reasonable to expect to find more papers using this embedding technique, as the traditional inclusion in paraffin is now accompanied by inclusion techniques in high- and low-temperature hard resins. Results instead showed that mechanical tests, mainly performed by indentation tests, are usually carried out on fresh or frozen/thawed samples, before histological processing. Specific shrewdness to avoid tissues damages and bias that could alter the subsequent analyses was applied, by keeping samples in saline solution with specific pH and temperature, or, alternatively, performing the test in the region of interest different from that employed for histology. Other technical aspects frequently reported are the stable fixation of samples, for example, with screws or cement, to guarantee the maintenance of the position of the sample perpendicular to the indenter as much as possible. In fact, one of the most critical aspects in the execution of indentation tests on fresh samples is the correct alignment with the machine, which is clearly easier when samples are already embedded in hard resin. Moreover, resin embedding allows the analysis of the biomechanical competence also of the subchondral and trabecular bone underlying articular cartilage, that in the reviewed papers always lacks. The great prevalence of indentation tests among the mechanical tests available provides a starting point for reflection on the use of this technique in the evaluation of OC regeneration. This method, in fact, has proven to be precise in evaluating tissue deformation and mechanical properties of regenerated tissues, in comparison to native ones, with possibilities to deepen the analyses at micro- and nanolevel [76]. It is reasonable to think that the improvement of these biomechanical tests as well as the progress in the level of investigation at the nanometric level will provide fundamental indications over time not only in terms of optimization of the design and production of scaffolds and materials for OC regeneration but also in relation to the anatomical site involved.

**Figure 2:**
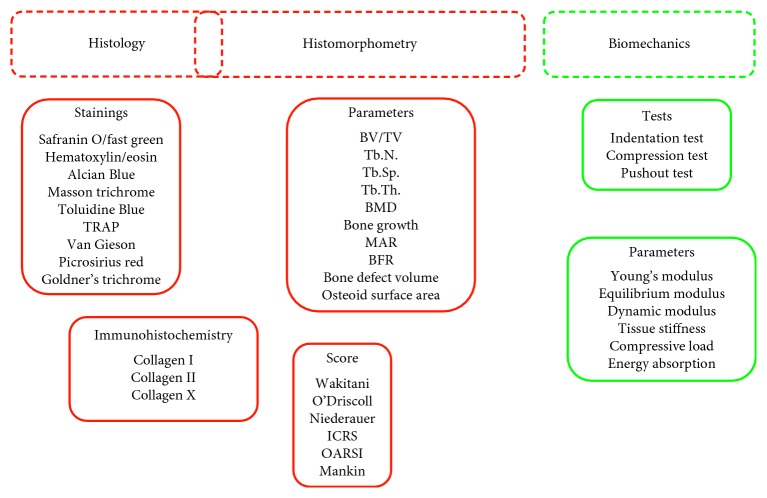
Overview of methods employed for histological, histomorphometrical, and biomechanical evaluations.

**Figure 3:**
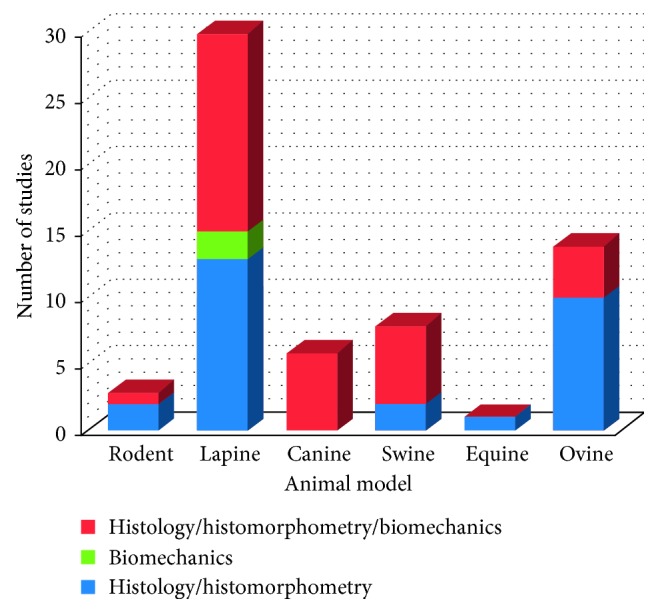
Number of studies in relationship with the animal model employed in which histology and/or histomorphometry and/or biomechanics are performed.

Histomorphometry is often performed on 2D paraffin or resin-embedded specimens, different from microCT that is performed on 3D volumes. The cutting plan of the histological sections is not always clearly indicated in the papers, and it is not easy to extrapolate this information from the observation of the images, sometimes acquired at high magnification. However, the most used planes are the sagittal and frontal planes, which allow to observe the osteochondral region in its entirety, both at the level of the cartilage and of the bone. In most of the studies, it is not reported on how many slices 2D histomorphometry has been performed. If 2D morphometry is not carried out on a relevant number of sections representative of the entire defects, this might cause the lack of arising of statistically significant differences among experimental groups. Moreover, data extracted from reviewed articles show a general troubling lack of statistical analyses to evaluate the number of animals to be employed, in relationship with experimental groups, animal model, number of implants, and experimental times. An a priori power analysis to exactly define the numerical consistency of animal groups required to reach the statistical significance is imperative in primis for ethical concerns, secondly to achieve correctly the scientific objectives with related costs (Figures 2 and 3).

**Figure 4:**
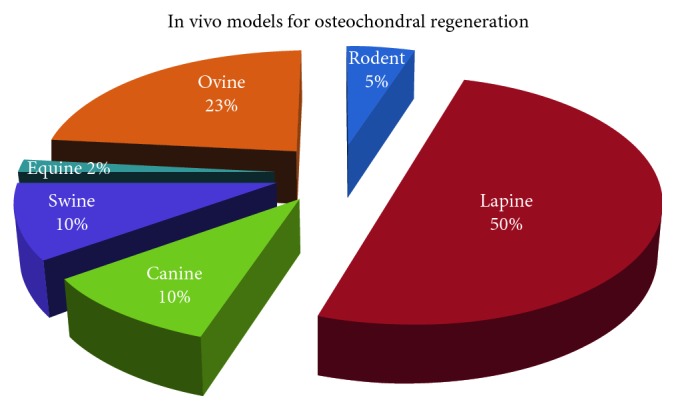
Distribution (%) of in vivo models employed in the reviewed studies for osteochondral regeneration.

As far as the selected animal model is concerned, rodent result is not widely used in the literature probably because of the small dimensions, making it difficult to perform all of the above biomechanical analysis of indentation rather than histomorphometric or microtomographic ones. Despite the reduced costs of management and the availability of different strains, as well as the possibility to set up allogenic or xenogenic models, the small size of the joint and thinnest articular cartilage make their use more difficult and far from the clinical scenario. Similarly, studies involving equine models are infrequent, but in this case, the reason might be related to the demanding management of such big animals, above the ethical and affective concerns. This last aspect is particularly felt also for the canine model whose use, despite its potentiality due to the spontaneous development of joint diseases similar to humans, is not allowed in some countries. In addition, the horse is unable to maintain protected weight-bearing protocols [77]. Therefore, the location of the defect should be carefully considered to avoid early overloading. However, the thickness of equine cartilage similar to humans' and the low regeneration ability, as well as the possibility to perform specific analyses as arthroscopy, make this model quite fascinating. Porcine and ovine models share with equines some advantages, namely, the joint size, cartilage and subchondral bone thickness, accessibility for arthroscopic procedures, and limited intrinsic healing capacity. However, their management and costs can be quite challenging [77]. The most used animal model is still the rabbit, because its dimensions represent a good compromise for greater ease of management and costs and the possibility of obtaining sufficiently large anatomical segments. However, two critical aspects of this model should not be overlooked: the peculiarity of the animal's load, which makes it very different from that of humans, and its great regenerative capacity, which keeps a debate open with continuous updates on the correct size of the defects to be created to properly evaluate bone regeneration (Figure 4).

Moreover, the overview of the animal models employed shows troubling inhomogeneities in terms of defect dimensions. This observation brings to light a very common controversial aspect in regenerative medicine studies, which makes it difficult to perform an easy comparison between studies and to properly define a defect as “critical.” As mentioned above, animal cartilage thickness is greatly different from that of humans (2-3 mm), with a difference of at least one millimetre in large models up to at least 2.5 millimetres in small animals. This is reflected in the dimension of the defects created, which should in any case be related to the experimental time adopted, which should not be less than 8 weeks for rabbits and 24 weeks for sheep, although longer experimental times are recommended [72]. According to these indications, the experimental times selected in the reviewed articles were coupled to the experimental models, being mostly 12 weeks for lapine and 24 weeks for canine, swine, and ovine models.

Additionally, the age of the animals must be taken into consideration since the regenerative abilities, as well as the effects of the load, are different depending on the age. A great variety has been observed in almost all animal models reviewed; perhaps, a greater homogeneity has been found only in the ovine model, in which age ranged between 2 and 4 years. Unfortunately, in many studies, age is not reported and “skeletally mature” is the only indication provided.

Apart from the Ho et al.'s study, there are no studies that compare the histological/histomorphometric and biomechanical characteristics of OC regeneration between areas subjected to high or low mechanical loads (such as femoral condyles as a high mechanical load area or as the trochlea as an area subjected to low mechanical load) in the same animal model [50]. This is important because it is known that the mechanical load has a positive effect on the repair/regeneration of both the chondral and bone tissues. Mechanical regulation of cartilage with the beneficial effects during cartilage regeneration have long been known [78]. The biomechanical environment of articular cartilage and subchondral bone, consisting of compressive and shear stresses and hydrostatic and osmotic pressures, governs with appropriate magnitudes the development and homeostasis of the tissue, as well as its regeneration. Chondrocytes are able to differently respond to biomechanical stimulations [79]: hydrostatic pressure increases the expression of metabolism related factors and activates the expression of genes associated with various cellular processes, such as extracellular matrix synthesis (cartilage oligomeric matrix protein COMP, type II and IX collagen, and GAGs) [80, 81] and proinflammatory gene suppression [82].

Another valuable aspect is what kind of data mechanical results is compared with. In some papers, results obtained from treated lesions were compared with the contralateral untreated limb or with data from healthy tissue. Another trend found is performing the evaluation on the adjacent native tissue, especially for cartilage. However, it must be considered that, though close but not within the treated region, they still suffer from the effects related to surgical and treatment procedures and the consequent alterations of the load by the animal. In fact, some works have observed in the areas adjacent to those treated mechanical characteristics halfway between those of the regenerated tissue and the healthy one [9].

Many reviews in the literature have addressed the topic of the most used animal models or current trends in treatments for OC defects, but to our knowledge, there are none that focuses on the type of evaluation performed.

In recent years, very different studies have been carried out; all in all, some points can be focussed and reference points were considered for the development of new studies. Among histological techniques, paraffin embedding combines the possibility to perform the evaluation of the cellularity and structure of cartilage and subchondral bone, thanks also to the better yield of stainings such as Safranin O, which are able to highlight fundamental histological aspects for assessing osteochondral regeneration. Although the existing scores have many criteria in common, it is advisable to combine more than one to cover all the aspects necessary for the evaluation of both the cartilaginous and the bony district. Among all scores, the most complete still scores are O'Driscoll score and Sellers score. The immunohistochemical quantification of the collagen component remains indispensable, and it would be important to place it with assessments of subchondral bone in terms of bone metabolism/regeneration, among the evaluation of architecture with imaging methods. Finally, more effort should be put for biomechanical tests to assess the quality of regenerated cartilage, in terms of load absorption capacity, stiffness, etc., as well as the evaluation of microhardness techniques for the evaluation of the mechanical competence of the subchondral bone, a field still little explored.

The use of comparable methods and the standardization of protocols for biomechanical tests will be essential to make the results obtained by such studies comparable and more reliable.

## Figures and Tables

**Table 1:** Data extraction of papers involving small-medium animal models.

Experimental model	Anatomical site (defect dimension) and experimental time	Osteochondral treatment	Histological, histomorphometric, and biomechanical methods	Main outcomes	Author
*Rodent model*					
Fifty-five rats (6 weeks old)	Cartilage defect (2 mm Ø and 1 mm depth) in the patellar groove for 1 and 2 months	Bilayered collagen scaffold with or without hESC-MSC	(i) ICRS score (ii) Paraffin embedding (iii) H&E and Safranin O stainings (iv) Indentation test on fresh explants submerged in PBS: Young's equilibrium modulus	Similar trends between the histomorphometric score and biomechanical analysis	Zhang et al. [10]

Nine male athymic nude rats (11 weeks old)	Critical-size defects in the trochlear groove (1.4 mm Ø and 1 mm depth) for 2 months	Micromasses of hPDCs with or without TGF-*β*1	(i) Paraffin embedding (ii) Alcian Blue staining (iii) IHC: Col I, Col II, nuclei, and lubricin (iv) MicroCT: BV/TV, Tb.Th., Tb.Sp., and Tb.N.	MicroCT showed heterogeneous regeneration across the defects	Mendes et al. [11]

Fifty male Wistar rat (4 months old)	Defects in the trochlear groove (1.5 mm Ø and 1.5 mm depth) for 2 months	MeHA hydrogel seeded with MSCs or chondrogenically primed MSCs cultivated either free loading or dynamically compressed	(i) Paraffin embedding (ii) Wakitani score (iii) Safranin O staining (iv) IHC: Col II	Dynamic compression and chondrogenic priming synergistically improved regenerative properties of MSCs	Lin et al. [12]

*Lapine model*					
Twelve young adult NZW rabbits	Defects in the weight-bearing areas of femoral condyles (4 mm of chondral defect followed by a 2 mm hole in the centre of the 4 mm defect) for 3 months	ADM alone (rabADM) or in association with IPFP-MSCs (cells + rabADM)	(i) Paraffin embedding HC: Col I and II (ii) Quantification of the total area of cartilage repair by 2D analysis	Significant differences in type II collagen staining	Ye et al. [13]

Ten NZW male rabbits (5 months old)	Defects in the medial femoral condyles (4 mm Ø and 4 mm depth) for 40 days	Collagen scaffold alone or seeded with rabbit BMC; half of the animals stimulated by PEMFs	(i) Niederauer score (ii) Paraffin embedding (sagittal cut) (iii) Safranin-O/fast green staining (iv) Modified O'Driscoll score quantification of new cartilaginous tissue over and under the tidemark	Significant effects in Niederauer and O'Driscoll scores and in percentage of cartilage	Veronesi et al. [14]

Twelve female skeletally mature NZW rabbits	Defects in the central medial femoral condyles (3.5 mm Ø and 2 mm depth) for 1.5 months	Bilayered collagen type I/III scaffold seeded with either culture-expanded allogenic chondrocytes (ACI-CHDR) or synovium-derived stem cells (ACI-SMSC)	(i) ICRS subscore and OARSI score (ii) Paraffin embedding (sagittal cut) (iii) H&E and Safranin O staining (iv) IHC: Col II, X (Remmele–Stegner score) (v) Indentation test on fresh samples: cartilage thickness, instant modulus, and shear modulus	Similar trends among instantaneous and shear modulus and OARSI score	Schmal et al. [15]

Sixteen male NZW rabbits (34 weeks old)	Defects in the patellar groove (3 mm Ø and 2–2.5 mm depth) for 3 months	3-dimensional constructs fabricated using Col II hydrogel alone (Col II) or associated with auricular chondrocytes (AU-Col II)	(i) Modified ICRS score (ii) Paraffin embedding (sagittal cut) (iii) H&E, Masson's trichrome, and Alcian Blue stainings	Significance in histological scores and defect healing	Wong et al. [16]

Ten male NZW rabbits (10 months old)	Full-thickness cartilage defects in the patellar groove (4 mm in Ø and 3 mm in depth) for 3 weeks	Autologous BMSCs seeded on type I collagen scaffold in association or not with LLLT	(i) Paraffin embedding (sagittal cut) (ii) H&E staining (iii) Quantification of new cartilage formation, new bone formation%, measure of inflammation	No significant difference in new cartilage formation and inflammation; significance in new bone formation	Fekrazad et al. [17]

48 NZW rabbits (6–8 months old)	Defects in the trochlear groove (4 mm Ø and 3 mm depth) for 3 and 9 months	Regenerated silk fibroin scaffold alone (SF) or seeded with autologous chondrocytes (SFC); fibrin glue containing autologous chondrocytes (FGC)	(i) Wakitani score (ii) Paraffin embedding (sagittal cut) (iii) Modified O'Driscoll, Keeley and Salter score (iv) H&E, Alcian Blue and Masson's trichrome stainings (v) IHC: Col II (vi) Indentation test on fresh samples: ultimate compressive strength (UCS) and compressive Young's modulus	Significant differences in histological scores but not in biomechanical data	Kazemnejad et al. [18]

Twenty-eight female skeletally mature NZW rabbits	Defects in the medial femoral condyle (4 mm Ø and 5 mm depth) for 13 weeks	Autologous BMP‐2-activated muscle tissue directly implanted into OC lesions	(i) Paraffin embedding (ii) Extended O'Driscoll score (iii) Safranin O/fast green stainings (iv) IHC: Col I and II (v) Quantification of bone area within the subchondral defect (v) Indentation test on fresh samples: stiffness	Similar trends between the bone area quantification and biomechanics	Betz et al. [19]

Forty-one skeletally mature NZW rabbits	Full-thickness defects in the femoral groove (5 mm Ø and 6 mm depth) for 1, 2, and 6 months	Combined material comprising a scaffold-free tissue-engineered construct (TEC) derived from synovial mesenchymal stem cells and hydroxyapatite (HA) artificial bone (TEC-HA) Control group: HA artificial bone	(i) Paraffin embedding (ii) O'Driscoll score (iii) H&E and Toluidine Blue staining (iv) Microindentation test (at 6 months): stiffness	Significance in the histological score but not in biomechanics	Shimomura et al. [20]

Nine skeletally mature male NZW rabbits	Defects in the medial femoral condyles (2.7 mm Ø and 4.0 mm depth) for 6.5 months	(i) Poly(1,8-octanediol-co-citrate) (POC) with 60 weight % hydroxyapatite nanocrystals (POC-HA) (ii) Poly-L-lactide (PLL)	(i) Paraffin embedding (longitudinal cut) (ii) Niederauer score (iii) Masson–Goldner trichrome staining (iv) Quantification of total area and range of depth of tissue ingrowth, active osteoid surface area/total trabecular bone surface area, total osteoid surface area/total trabecular bone surface area, and trabecular bone surface area/total tissue area (v) Measurement of fibrous capsule widths	No significant differences in all histomorphometric evaluations	Chung et al. [21]

Seven male and female NZW rabbits (13 or 32 months old)	Defects in the trochlear groove (1.5 mm Ø and 2 mm depth drill holes) for 70 days	(i) 10 kDa chitosan/blood implant with fluorescent chitosan tracer (ii) 40 kDa chitosan/blood implant with fluorescent chitosan tracer	(i) Modified O'Driscoll score (ii) Paraffin embedding (sagittal cut) (iii) SafO staining (iv) IHC: Col I and II (v) Quantification of total chondral repair tissue area, (including bone overgrowth); percentage SafO, Col-1- or Col-2-positive-stained tissue (excluding bone overgrowth) (vi) MicroCT on fresh samples: Residual hole depth and residual hole area below the surface	Significant differences in bone morphometry and O'Driscoll scores	Guzmán-Morales et al. [22]

Five male and female NZW rabbits (30-months old)	Defects in the trochlea (microdrill hole defects, 1.4 mm Ø, and 2 mm depth) for 1 and 21 days	150, 40, and 10 kDa chitosan solutions, mixed with autologous rabbit whole blood and clotted with tissue factor	(i) MicroCT on fresh samples: residual hole depth and residual hole area below the surface (ii) Paraffin embedding (sagittal cut) (iii) SafO staining (iv) IHC: Col I and II (v) Quantification of GAG, col I or col II (%); distribution of repair tissues in treated defects and volume density of neutrophils and stromal cells (vi) TRAP quantification	Significant differences in microCT, GAG, col II, and col I quantifications and volume density of neutrophils	Lafantaisie-Favreau et al. [23]

Twenty NZW rabbits (18 weeks old)	Defects in the weight-bearing area of medial femoral condyles (3 mm Ø and 3 mm depth) for 2 weeks and 1, 2, and 4 months	Allogeneic scaffold-free bioengineered chondrocyte pellet (BCP)	(i) Paraffin embedding (sagittal cut) (ii) Modified O'Driscoll score (iii) H&E, Safranin O/fast green staining (iv) IHC: Col I and II, type I and type II (v) PCNA stainings (vi) Quantification of % area filled in defect, cartilage thickness, and bone area	Significant differences in the score and cartilage thickness	Cheuk et al. [24]

Forty NZW rabbits (12–15 months old)	Defects in the weight-bearing area of medial femoral condyles (2 mm Ø with 1–1,5 mm depth) for 2 and 4 months	Osteochondral defect (acute osteoarticular injury)	(i) Paraffin embedding (sagittal cut) (ii)Manking score (iii) Safranin O/fast green staining (iv) Sagittal-plane laxity measurement (at 8 and 16 weeks) (v) Contact stress test on 7 fresh cadaver knees	Significance in the histological score	Vaseenon et al. [25]

Fourty-two adult male NZW rabbits	Defects in the patellar groove (4 mm Ø and 3.5–4 mm depth) for 1.5 and 3 months	Bilayered microporous scaffold with collagen and electrospun poly-L-lactic acid nanofibers (COL-nanofiber) and bilayer COL scaffold, seeded with BMSCs	(i) ICRS score (ii) Paraffin embedding (iii) H&E and Safranin O/fast green staining (iv) Indentation test (at 12 weeks): Young's moduli on fresh samples placed in PBS at room temperature before testing. (v) microCT: subchondral bone	Similar trend between histological scoring system and biomechanical test	Zhang et al. [26]

Nine female NZW rabbits (6 months old)	Defects in the medial femoral condyle (4 mm Ø and 4 mm depth) for 3 months	(i) 70/30 poly(ethylene oxide terephthalate)/poly(butylene terephthalate) (PEOT/PBT) scaffold (ii) 55/45 PEOT/PBT	(i) Histological scoring system (O'Driscoll score) on 2-hydroxyethyl methacrylate (Technovit) embedded samples (thionine staining) (midsagittal cut)	Significance in the histological scoring system	Jansen et al. [27]

Forty-eight NZW rabbits (7 months old)	Defects in the patellar groove (5 mm Ø and 10 mm depth) for 2 weeks and 1, 2, and 4 months	OC defects treated with low-level He-Ne laser therapy (LLLT) 3 times a week	(i) Paraffin embedding (sagittal cut) (ii) H&E, Toluidine Blue staining (iii) Pineda score	Significant acceleration of healing at 4 and 6 weeks	Bayat et al. [28]

20 adult male NZW rabbits	Defects in the femoral epiphysis (6 mm Ø and 8 mm depth) for 2 months	Mineralized HA-alginate scaffold compared to a commercially available collagen-hydroxyapatite composite scaffold	(i) Niederauer score (ii) PMMA embedding (sagittal cut) (iii) Stevenel Blue/van Gieson pichrofucsin staining (iv) Quantification of MAR and BFR (v) MicroCT on fresh samples: defect BV/TV; defect Tb.Th.; defect Tb.N.; defect Tb.Sp.; peri-implant BV/TV; peri-implant Tb.Th., Tb.N., and Tb.Sp.	Significance in microCT evaluations and not in dynamic morphometric analyses	Filardo et al. [29]

Sixty skeletally immature male NZW rabbits (3 months old)	Full-thickness defects in the trochlear groove (4 mm Ø and 4 mm depth) for 2 and 9 months	Autogenous periosteal grafts under the influence of (i) group a— active intermittent motion (AIM), euthanized at 8 weeks; group B— continuous passive motion (CPM), euthanized at 8 weeks; group C—AIM, euthanized at 36 weeks; (ii) Group D—CPM, euthanized at 36 weeks	(i) Indentation test on fresh samples: elastic stiffness (ii) paraffin embedding (sagittal cut) (iii) O'Driscoll score (iv) H&E, Masson trichrome, and Alcian Blue staining (v) Quantification of thickness and area of the regenerated tissue; thickness of the normal cartilage surrounding the defect	Significance in thickness of regenerated tissue and in elastic stiffness	Martin-Hernandez et al. [30]

Forty-two male NZW rabbits (7 months old)	Full-thickness defects in the patellar groove (5 mm Ø and 10 mm depth) for 2 and 1, 2, and 4 months	OC defects treated with low-level He-Ne laser therapy (LLLT) 3 times a week	(i) Indentation test on previously frozen samples: instantaneous stiffness, maximum force, equilibrium load, and energy absorption	Significance only in the energy absorption	Javadieh et al. [31]

Twenty mature female NZW rabbits	Defects in the medial femoral condyle (2.5 Ø and 3 mm depth) for 1, 2, and 3 months	OC defects treated with low-dose irradiation	(i) Paraffin embedding (ii) O'Driscoll score (iii) H&E and Safranin O staining (iv) Indentation test on previously frozen samples: cartilage stiffness	No statistical significance was seen in any parameter	Öncan et al. [32]

Thirty-four male NZW rabbits	Full-thickness defects in the medial and lateral femoral condyles (3 mm Ø and 3 mm in depth) for 6 and 12 weeks	Poly(lactic-co-glycolic acid) with or without fibrin as cells carrier: (i) PLGA/Fibrin/BMSCs (PFC group) (ii) PLGA/BMSCs (PC group)	(i) ICRS score (ii) Paraffin embedding (iii) H&E; Alcian Blue; Safranin O staining (iv) IHC: Col II (v) Cartilage-specific gene expression (vi) Quantification of sGAG (v) Compression test (at 12 weeks)	Similar significant trends in histological score, GAG content and biomechanical strength	Rahman et al. [33]

Thirty-five skeletally mature NZW rabbits (24 weeks old)	Full-thickness defects in the patellar groove (5 mm Ø and 6 mm depth) for 1, 2, and 6 months	(i) Combined material: bTCP-based hybrid implant coupled with a scaffold-free tissue-engineered construct (TEC) derived from synovial mesenchymal stem cells (TEC/bTCP) (ii) Scaffold-free tissue-engineered construct (TEC) derived from synovial mesenchymal stem cells and hydroxyapatite (HA) artificial bone (TEC/HA)	(i) Histological grading system (resurfacing:0–2) for gross examination (ii) Paraffin embedding (iii) Modified O'Driscoll score (iv) H&E and Toluidine Blue staining (v) Microindentation test: tissue stiffness	Similar trends among cellular morphology, total histological score, and biomechanics	Shimomura et al. [34]

Five NZW rabbits (5–6 months old)	Defects in the trochlear groove (3 mm Ø and 2 mm depth) for 3 and 6 months	Cell carrier prepared from articular cartilage slices, designated cartilage extracellular matrix- (ECM-) derived particles (CEDPs) seeded with rabbit ACs or ASCs	(i) ICRS score (ii) Paraffin embedding (iii) H&E, Toluidine Blue and sirius red staining (iv) OARSI score (v) IHC: Col I and II (vi) Nanoindentation tests on fresh samples (6 months): hardness, contact stiffness and reduced modulus (vii) MRI: cartilage regeneration (viii) microCT: Tb.Th. and BV/TV	Same significant trend in histological, microCT, and biomechanical evaluations	Yin et al. [35]

Eighteen NZW rabbits (15 weeks old)	Defects in the medial and lateral femoral condyles (3 mm Ø and 3 mm depth) for 2, 4, and 6 months	Expandable gelatin scaffold seeded with rabbit chondrocytes (C + S group) compared to OC defects treated with allogenic chondrocyte injection (positive control), scaffold alone (S) and empty defect	(i) O'Driscoll score (ii) Paraffin embedding (iii) H&E, Alcian Blue stainings (iv) Quantification of integration, apposition, and disintegration of regenerated tissue (v) IHC: Col I, II, and X and S-100 (vi) Compression test on fresh samples	Similar trend among the macroscopic score, histomorphometry, and compressive strength at each time point	Wang et al. [36]

Twenty-seven NZW rabbits (3 months old)	Full-thickness defects in the trochlea (4 mm Ø and 4 mm depth) for 6, 12, or 24 weeks	Oriented bovine cartilage ECM-derived scaffold using thermal-induced phase separation (TIPS) technology and seeded with rabbit BMSCs: (i) cell-oriented scaffold construct; (ii) cell-random scaffold composite	(i) Paraffin embedding (ii) H&E, Toluidine Blue, and Safranin O staining (iii) Modified O'Driscoll score (iv) microCT (v) Unconfined compression test (UCC) on fresh samples: Young's modulus (v) Quantification of total DNA level, total GAG, and collagen content	Similar trends among histomorphological score, DNA, GAG, and collagen content and biomechanics	Jia et al. [37]

Fifty-two Japanese white rabbits (6 months old)	Defects in the trochlea (4.3 mm Ø and 7 mm depth) for 1, 2, 4, and 12 weeks	Hydroxyapatite- (Hap-) coated double-network (DN) hydrogel (HAp/DN gel)	(i) MMA embedding (sagittal cut) (ii) Villanueva bone staining (iii) IHC: procollagen 1A1 (iv) Pushout test (v) MicroCT: Bonding area and tissue density	Similar trend between microCT and biomechanics	Wada et al. [38]

Five female Japanese white rabbits (6 months old)	Defects in the trochlea (4.7 mm Ø and 7 mm depth) for 1 month	(i) Collagen fibril-based tough hydrogels based on the double network (DN) concept using swim bladder collagen (SBC) extracted from Bester sturgeon fish (SBC/PDMAAm) (ii) Hydroxyapatite- (Hap-) coated gel (HAp/c-SBC(ge-1)/PDMAAm)	(i) Pushout and compression test on fresh samples	Significant differences in biomechanical performance	Mredha et al. [39]

Forty-eight adult male NZW rabbits	Defects in the medial femoral condyles (4 mm Ø and 5 mm depth) for 1, 2, and 4 months	Porous tantalum (PT) loaded with BMP-7 (MPT group)	(i) SEM analysis (ii) MMA embedding (longitudinal cut) (iii) Toluidine Blue staining (iv) MicroCT (at 16 weeks): bone intertrabecular space (trabecular spacing, Tb. Sp); bone density; Tb.Th.; Tb.N.; BV/TV; (v) Launch test	Similar trend among histological grading system, micro CT, and biomechanics	Wang et al. [40]

Thirty-six skeletal mature NZW rabbits (5-6 months old)	Defects in the central medial femoral condyle (4 mm Ø and 5 mm depth) for 4 months	Bilayered PLGA/PLGA-Hap composite scaffold preseeded with BMSCs	(i) Paraffin embedding (longitudinal cut) (ii) H&E, Toluidine Blue, and Safranin O stainings (iii) IHC: Col II (iv) MicroCT on fresh samples (v) AFM test of Young's modulus and surface roughness (vi) Western blot: p-smad 1, p-smad 2, and Col I and II	Significant differences in protein expression but not in all other parameters	Xiangyu et al. [41]

Forty-two NZW rabbits (6–12 months old)	Defects in the trochlea (5 mm Ø and 5 mm depth) for 6 months	(i) Osteochondral allografts (OCA) stored in Tsmu (ii) OCA after vitrification	(i) Paraffin embedding (ii) Mankin score (iii) H&E, Safranin O/fast green staining (iv) Quantification of chondrocyte viability (fluorescein diacetate and ethidium bromide staining), proteoglycan (PG) type II collagen (v) Compression test on fresh samples: Young's modulus	Similar trends among gross score, chondrocyte viability, PG content, type II collagen, and Young's modulus	Cao et al. [42]

Ø = diameter; IHC = immunohistochemistry; Col = collagen; hPDCs = human periosteum-derived progenitor cells; TGF-*β*1: transforming growth factor *β*1; BV/TV = bone volume/trabecular volume; Tb.Th. = trabecular thickness; Tb.Sp. = trabecular separation; Tb.N. = trabecular number; microCT = microcomputed tomography; hESC-MSC = human embryonic stem cell-derived mesenchymal stem cells: PBS = phosphate-buffered saline; MeHA = methacrylated hyaluronic acid; NZW = New Zealand white; ADM =  acellular dermal matrix; IPFP = infrapatellar fat pad; H&E = hematoxilyn and eosin; BMC = bone marrow concentrate; HA = hydroxyapatite; PEMFs = pulsed electromagnetic field; BMP‐2 = bone morphogenic protein-2; OC = osteochondral, BMSCs = bone marrow mesenchymal stem cells; LLLT = low-level laser therapy; mar = mineral apposition rate; BFR = bone formation rate; AFM = atomic force microscope; sGAG =  sulphated glycosaminoglycan; bTCP = beta-tricalcium phosphate; ACs = articular chondrocytes; ASCs = adipose-derived stem cells; Tsmu = Taishan Medical University solution.

**Table 2:** Data extraction of papers involving large animal models.

Experimental model	Anatomical site (defect dimension and experimental time)	Osteochondral treatment	Histological, histomorphometric, and biomechanical methods	Main outcomes	Author
*Canine model*					
Twelve male dogs	Defects (11 mm Ø and 10 mm depth) in the load-bearing area of the femoral head for 3 and 6 months	Allogeneic BMSC-seeded DCM/DCBM scaffolds	(i) MicroCT on fresh samples: bone volume fraction (ii) Indentation test on fresh samples: stiffness	Similar trend between microCT and biomechanics (stiffness)	Qiang et al. [43]

Eight mongrel dogs	Defects (3.5 and 4.5 mm Ø and 10 mm depth) in the medial femoral condyle for 12 months	Autograft and allograft plugs	(i) Paraffin embedding (sagittal cut) (ii) Histological scoring system for proteoglycan content (iii) H&E and Safranin O stainings; (iv) MRI: MOCART score and T2 mapping (v) Indentation test on fresh samples submerged in saline solution: second shear modulus	No statistical significance was seen in any parameter	McCarty et al. [44]

Twenty-seven TOYO beagles (15 months old)	Defects in the patellar groove (5.0 mm Ø and 2.0 mm depth) for 27 weeks	Ultrapurified alginate gel with or without microfractures	(i) Paraffin embedding (longitudinal cut) (ii) Niederauer score (iii) H&E and Safranin O stainings (iv) IHC: Col I and II (v) GAGs content (vi) Changoor score for collagen orientation (vii) MicroCT on frozen samples: volume of mineralized bone (viii) Indentation test on fresh samples submerged in saline solution: stiffness	Similar trends between the histological and collagen orientation scores and biomechanical analysis of stiffness. No differences in microCT	Baba et al. [45]

Twelve male dogs (2 year-old)	High load bearing surface of femoral condyles (4.2 mm Ø and 6 mm depth) for 3 and 6 months	Decellularized OC construct with or without 1 × 10^6^ chondrogenically induced BMSCs	(i) Paraffin embedding (ii) Solchaga score on paraffin-embedded samples (H&E and Toluidine Blue stainings); quantification of glycosaminoglycan content (iii) MicroCT on fixed samples: BVF and BRP (iv) Indentation test on samples fixed with cement and submerged in saline and EDTA solution: stiffness of cartilage and of subchondral bone (only at 6 months)	Significant on histological score and not in other parameters	Yang et al. [46]

Sixteen female mongrel dogs (2–5 years old)	Defects (8 mm Ø and 8 mm depth) in the weight-bearing areas of the lateral and medial femoral condyles for 6 months	Allograft plugs stored in different storage media and temperature	(i) Paraffin embedding (ii) OARSI score (iii) H&E, Toluidine Blue, and picrosirius red stainings (iv) GAGs and collagen contents (v) Indentation test on thawed samples: instantaneous tissue and dynamic modulus	Significance in histological score and not in biomechanics	Cook et al. [47]

Sixteen female mongrel dogs (2–5 years old)	Defects (8 mm Ø and 8 mm depth) in the weight-bearing areas of the lateral and medial femoral condyles for 6 months	Allograft plugs stored in different storage media and temperatures	(i) Paraffin embedding (ii) OARSI score (at 1 week and 6 months) (iii) H&E, Toluidine Blue, and picrosirius red stainings (iv) Quantification of GAGs and collagen (v) Indentation test on thawed samples: instantaneous tissue modulus and dynamic modulus (at 6 months)	No statistical significance was seen in any parameter	Cook et al. [48]

*Swine model*					
Sixteen pigs (6 months old)	Defects (10 mm Ø, 4 mm depth) in the weight-bearing area of medial and lateral femoral condyles for 6 months	PGA/PLA scaffolds seeded with autologous BMSCs and cultivated in vitro for 2, 4, or 8 weeks	(i) Paraffin embedding (longitudinal cut) (ii) Wakitani and Pineda scores (iii) H&E, Safranin O, and sirius red staining (iv) IHC: Col I, Col II, and osteocalcin (v) Quantification of collagen and GAGa contents (vi) Indentation test on fresh samples: compressive load-displacement curve and Young's modulus	Similar trend between histological score and biomechanics	He et al. [49]

Yorkshire Duroc pigs (six months old)	Critical sized defects in the medial condyle and patellar groove (8 mm and 8 mm depth) for 6 months	Biphasic construct made of PCL for cartilage and PCL-TCP for bone with or without BMSCs	(i) Paraffin embedding (longitudinal cut) (ii) O'Driscoll score (iii) H&E, Toluidine blue/Safranin O, and Masson's trichrome stainings (iv) IHC: Col I and II (v) MicroCT on fresh samples: degree of mineralization (vi) Indentation test on thawed samples: Young's modulus	Inferior healing in the patellar groove than in medial condyle; similar trends and positive correlation between microCT and biomechanical tests for all groups at both locations	Ho et al. [50]

Twelve male Gottingen minipigs (19.8-months old)	Critical sized defects in the medial and lateral trochlear facets (6 mm Ø and 8 mm depth) for 6 and 12 months	Autologous bone graft with or without autologous cartilage chips	(i) Resin embedding (ii) ICRS II score (iii) H&E staining (iv) Quantification of hyaline tissue, fibrocartilage, fibrous tissue, bone, bone marrow and blood vessel area (v) MicroCT on fresh samples: bone defect volume	Histomorphometric parameters showed differences between groups (articular cartilage, fibrocartilage, fibrous tissue, and ICRS II); microCT showed significant differences between experimental times but not between experimental groups	Christensen et al. [51]

Eight female Goettingen minipigs (1.5–2 years old)	Defects (5.4 mm Ø and 8 mm depth) in the trochlear groove for 2 months	Collagen type I/III membrane with or without autologous BMSCs	(i) Paraffin embedding (longitudinal cut) (ii) O'Driscoll score (iii) Safranin O and col II stainings (iv) IHC: Col II	Better significant results in the O'Driscoll score	Jung et al. [52]

Eighteen Göttingen minipig (1.5–2.5 years old)	Critical size defects (6.3 mm Ø and 10 mm depth) in the trochlear groove for 1.5, 3, and 13 months	Autologous osteoperiosteal bone plug with or without subperiosteal injection of a chondrogenic and osteogenic growth factor mixture	(i) Paraffin embedding (sagittal cut) (ii) Safranin O staining (iii) ICRS II score (iv) Indentation test on fresh samples: compressive load-displacement curve	No statistical significance was seen in any parameter	Gotterbarm et al. [53]

Eighteen minipigs (7-8 months old)	Defects (7 mm Ø, 8 mm depth) in the medial femoral condyles for 6 months	PLGA scaffold with or without autologous chondrocytes or BMSCs	(i) Paraffin embedding (longitudinal cut) (ii) ICRS score (iii) H&E and Safranin O staining (iv) MRI: MOCART score and biomechanical properties (collagen matrix and hydration) (v) Indentation test on fresh samples: compressive modulus	Similar trend among histomorphometric, MRI scores (ICRS and MOCART), and biomechanics (compressive modulus)	Zuo et al. [54]

*Equine model*					
Five mature ponies	Defects (13 mm Ø and 7 mm depth) in femoral condyles with an inner hole (2.5 mm Ø and 10 mm depth) for 3, 6 (MRI and CT), and 13 months (microCT and histology)	Ad-BMP2 or Ad-BMP6 or Ad-GFP	(i) qMRI (ii) CT in vivo and microCT ex vivo: lesion area and BMD for the lesion, drill, and adjacent subchondral bone (iii) Paraffin embedding (iv) O'Driscoll score (v) H&E, Toluidine Blue and Safranin O stainings	Similar trends between MRI (T1 relaxation time) and clinical CT (BMD) at 12 weeks	Menendez et al. [55]

*Ovine model*					
16 adult female Dutch milk goats (4 years)	Defects (6 mm Ø and 6 mm depth) in each talus for 6 months	Demineralized bone matrix (DBM) with and without platelet-rich plasma (PRP)	(i) Paraffin embedding (longitudinal cut) (ii) microCT: BV/TV (iii) Goldner's trichrome and Toluidine Blue stainings (iv) Quantification of mineralized bone surface area and osteoid surface area (%), number of osteoclasts, osteoblasts, and osteocytes, MAR	No differences between groups	van Bergen et al. [56]

8 skeletally mature female Dutch milk goats	Osteochondral defects (5 mm Ø, 3.5 mm depth) were created in medial condyles and trochlear grooves for 1 and 4 months	Acellular collagen I/III scaffolds or scaffolds seeded with SVF cells or cultured ASCs	(i) Indentation test (fresh sample): 50, 100, 200, and 300 *μ*m indentation at a constant speed of 20 *μ*m/sec with 4 mm Ø bold tip probe (ii) Paraffin embedding (sagittal cut) (iii) H&E and Alcian Blue stainings (iv) IHC: COLLI, COLLII (v) microCT (vi) GAGs quantification	No significance in biomechanical test: better histological and immunohistochemical outcomes in acellular construct	Jurgens et al. [57]

Goat	Critical size defect 6 mm Ø × 6 mm depth in each medial femoral condyles for 6 and 12 months	(1) Maioregen scaffold (2) Articular cartilage and growth plate ECM from porcine hind limbs AC-GP-ECM-derived bilayered scaffold	(i) MicroCT (ii) Paraffin embedding (longitudinal cut) (iii) H&E, Safranin O, and picrosirius red stainings (iv) IHC: COLLII (v) ICRS score	Hyaline-like repair tissue, better collage fiber organization of repaired tissue, and parallel fiber orientation with a lower range of dispersion in the superficial cartilage region	Cunniffe et al. [58]

10 skeletally mature female Merino sheep (2–2.5 years)	Bilateral full thickness defects (4 mm Ø and 12 mm depth) created 2 mm below the calcified layer in the medial femoral condyles for 6 and 12 months	Triphasic implant engineered using *β*-tricalcium phosphate osseous phase and Coll I hydrogel chondral phase, with MSCs vs. autograft	(i) ICRS score (ii) O'Driscoll score (iii) Siebert semiquantitative score (iv) Toluidine Blue and Levai-Laczko stainings (v) Indentation test (maximum load 40 N) fresh sample (vi) IHC: COLLII (vii) MicroCT	No biomechanical differences between the groups	Marquass et al. [59]

28 female Merino sheep (2–4 years old)	7 mm Ø and 25 mm depth osteochondral defect in the centre of the load-bearing area of the medial femoral condyle for 1.5, 3, 6.5, and 13 months	Cylindrical plugs of microporous b-TCP (Ø: 7 mm; length: 25 mm; porosity: 43.5 ± 2.4%; pore Ø:∼5 *μ*m) seeded with autologous chondrocytes cultured for 4 weeks	(i) Paraffin embedding (sagittal cut) (ii) ICRS score (iii) ESEM (iv) TEM (v) MicroCT (vi) Masson's trichrome, Safranin O, Giemsa, and TRAP stainings (vii) O'Driscoll score (viii) IHC: COLLI, COLLII, COLLX, and ALP (ix) Quantification of mineralized bone substance and TCP proportion	Degradation of ceramic proportional to bone formation; new cartilage formation and integration, although not with the same values of native one	Bernstein et al. [60]

5 skeletally mature Arcott cross female sheep (2–4 years old)	Six 2 mm Ø, 2.5 to 8.5 mm deep Jamshidi biopsy holes were created bilaterally in the weight-bearing area of medial femoral condyle for 1 day, 3 weeks and 3 months	Presolidified chitosan-blood implant with fluorescent chitosan tracer	(i) Paraffin embedding (longitudinal cut) (ii) MicroCT (iii) Safranin O/fast green/iron hematoxylin, Gomori trichrome, and von Kossa/Toluidine Blue staining (iv) IHC: COLLI, COLLII	Bone plate-induced chondroinduction is an articular cartilage repair mechanism; Jamshidi biopsy repair takes longer than 3 months and can be influenced by subchondral chitosan-blood implant	Bell et al. [61]

24 adult goats (2–3 years old)	Osteochondral defect in the medial femoral condyles (6 mm Ø and 8 mm depth) for 3, 6, and 12 months	Multilayered scaffolds with oriented articular cartilage extracellular matrix- (ACEM-) derived cartilage layer, porous 3D printing (3DP) PLGA/TCP bone layer (BL), and an intermediate PLGA/TCP compact interfacial layer	(i) ICRS score (ii) Safranin O and Toluidine Blue staining (iii) O'Driscoll score (iv) IHC: collII (v) Compression test (initial load of 0.05 N, speed 0.01 mm/s)	MLS enhances hyaline-like tissue formation with better mechanical properties	Jia et al. S, 2018 [62]

6 crossbred adult sheep	Critical size osteochondral defect (7 mm Ø, 5 mm depth) in the medial and lateral femoral condyles for 6 months	Biphasic HA-HYA alginate- based scaffold (bony layer 1.25% alginate and 4% HA; chondral layer 1% alginate and 0.5% HYA)	(i) Fortier-modified score (ii) MicroCT: BV/TV; Tb.Sp.; Tb.Th.; and Tb.N. (iii) Paraffin embedding (sagittal cut) (iv) Safranin O/fast green staining (v) Pineda score (vi) IHC: COLLI, COLLII, VEGF	No differences were found between groups.	Filardo et al. [29]

14 skeletally mature goat	Osteochondral defect (6 mm Ø, 8 mm depth) in the medial and lateral femoral condyles for 6 months	Biphasic osteochondral scaffold prepared using coralline aragonite with 1 to 2 mm depth drilled channels in the cartilage phase (+HA impregnation) or in the bone phase	(i) Fortier-modified score (ii) ICRS score (iii) paraffin embedding (longitudinal cut) (iv) Safranin-HE, Masson trichrome, Safranin O/fast green stainings (v) IHC: COLLI and COLLII (vi) O'Driscoll score	Mechanical modification with drilled channels and impregnation of HA within the coral pores enhanced the scaffold's cartilage regenerative potential	Kon et al. [63]

12 skeletally matured female adult sheep	Osteochondral lesion (7 mm Ø, 9 mm thickness) in the right medial and lateral femoral condyles for 6 months	Osteochondral biomimetic scaffold with and without PRP	(i) Paraffin embedding (sagittal cut) (ii) Safranin O/fast green staining (iii) Niederauer score (iv) IHC: COLLII	HA-coll scaffold promotes regeneration even without PRO	Kon et al. [64]

22 Sardinian sheep (5.5 years old)	Bilateral osteochondral defects in medial and lateral condyles (6 mm Ø and 2 mm depth) involving subchondral bone for 1, 2, 6, 12, and 24 months	Embryonic stem-like (ESL) cells embedded in fibrin glue	(i) Indentation test (fresh samples) (ii) H&E and Safranin O staining (iii) Score by Kaplan (iv) IHC: COLLII (v) FISH	ESL cells enhance the regeneration of hyaline cartilage	Manunta et al. [65]

24 skeletally mature female merino-mix sheep	7.3 mm Ø defect and 12 mm in height in the central weight-bearing area of the femoral condyles for 3 and 6 months	Osteochondral autograft bottomed (recipient site depth 10 mm) and unbottomed (recipient site depth 12 mm)	(i) Paraffin embedding (sagittal cut) (ii) Safranin O/von Kossa, Safranin O/fast green stainings, and TRAP staining	Full graft support improves long-term integration	Nosewicz et al. [66]

12 female Merino sheep (2 years old)	Osteochondral defects in the weight-bearing area of femoral condyles (9.4 mm Ø and 1.1 cm depth) for 6 weeks	Biphasic scaffold of hydroxyapatite/collagen (scaffold a) and allogenous-sterilized bone/collagen (scaffold B) with or without chondroblasts	(i) ICRS score (ii) Paraffin embedding (sagittal cut) (iii) TRAP staining (iv) H&E and Toluidine Blue stainings (v) IHC: coll II and CD68 (vi) Gene expression: Col1A1, COLIIA1, SOX9, and CEP-68	More immunocompetent cells around scaffold and a higher expression of COLLII and SOX9 for scaffold B	Schleicher et al. [67]

28 female Merino sheep (2–4 years old)	Osteochondral defect of 7 mm Ø and 25 mm in height in the center femoral condyles for 1.5, 3, 6.5, and 13 months	Microporous beta TCP scaffold (7 mm Ø and 25 mm length) preseeded with autologous chondrocytes	(i) Indentation test in a special mount (3 mm Ø indenter, 200 *μ*m penetration, maximal load 1.5 N): achieved load, absorbed energy, and contact stiffness (ii) PMMA embedding (longitudinal cut) (iii) ICRS score	Mechanical properties of TCP scaffold were similar to native cartilage Lower score in the central area	Mayr et al. [68]

GAGs = glycosaminoglycan; OC = osteochondral; BMSCs = bone marrow-derived mesenchymal stem cells; BVF = bone volume fraction; BRP = bone regeneration percentage; PCL = polycaprolactone; PCL-TCP = olycaprolactone-tricalcium phosphate; Ad-BMP2 = adenoviral bone morphogenetic protein 2; Ad-BMP6 = adenoviral bone morphogenetic protein6; Ad-GFP = adenoviral green fluorescent protein; BMD = bone mineral density; DCM/DCBM = microfilaments of decellularized cartilage matrix/decellularized cancellous bone matrix; DMEM = Dulbecco's modified Eagle's medium; ECM = extracellular matrix; HA-HYA = hydroxyapatite-hyaluronic acid; DBM =  deminerilzed bone matrix; PRP = platelet rich plasma; PMMA= poly(methyl methacrylate); TCP = tetracalciumphosphate; SVF = stromal vascular fraction; ALP = alkaline phosphatase; PLGA = poly(lactic-co-glycolic acid).

## References

[1] Bowland P., Ingham E., Jennings L., Fisher J. (2015). Review of the biomechanics and biotribology of osteochondral grafts used for surgical interventions in the knee. *Proceedings of the Institution of Mechanical Engineers, Part H: Journal of Engineering in Medicine*.

[2] Widuchowski W., Widuchowski J., Trzaska T. (2007). Articular cartilage defects: study of 25,124 knee arthroscopies. *The Knee*.

[3] Andriolo L., Crawford D. C., Reale D. (2018). Osteochondritis dissecans of the knee: etiology and pathogenetic mechanisms. A systematic review. *Cartilage*.

[4] Niemeyer P., Feucht M. J., Fritz J., Albrecht D., Spahn G., Angele P. (2016). Cartilage repair surgery for full-thickness defects of the knee in Germany: indications and epidemiological data from the German Cartilage Registry (KnorpelRegister DGOU). *Archives of Orthopaedic and Trauma Surgery*.

[5] Sartori M., Pagani S., Ferrari A. (2017). A new bi-layered scaffold for osteochondral tissue regeneration: in vitro and in vivo preclinical investigations. *Materials Science and Engineering: C*.

[6] Tamaddon M., Wang L., Liu Z., Liu C. (2018). Osteochondral tissue repair in osteoarthritic joints: clinical challenges and opportunities in tissue engineering. *Bio-Design and Manufacturing*.

[7] Deng C., Chang J., Wu C. (2019). Bioactive scaffolds for osteochondral regeneration. *Journal of Orthopaedic Translation*.

[8] Dias I. R., Viegas C. A., Carvalho P. P., Oliveira J., Pina S., Reis R., San Roman J. (2018). Large animal models for osteochondral regeneration. *Osteochondral Tissue Engineering. Advances in Experimental Medicine and Biology*.

[9] Strauss E. J., Goodrich L. R., Chen C.-T., Hidaka C., Nixon A. J. (2005). Biochemical and biomechanical properties of lesion and adjacent articular cartilage after chondral defect repair in an equine model. *The American Journal of Sports Medicine*.

[10] Zhang S., Jiang Y. Z., Zhang W. (2013). Neonatal desensitization supports long-term survival and functional integration of human embryonic stem cell-derived mesenchymal stem cells in rat joint cartilage without immunosuppression. *Stem Cells and Development*.

[11] Mendes L. F., Katagiri H., Tam W. L. (2018). Advancing osteochondral tissue engineering: bone morphogenetic protein, transforming growth factor, and fibroblast growth factor signaling drive ordered differentiation of periosteal cells resulting in stable cartilage and bone formation in vivo. *Stem Cell Research and Therapy*.

[12] Lin S., Lee W. Y. W., Feng Q. (2017). Synergistic effects on mesenchymal stem cell-based cartilage regeneration by chondrogenic preconditioning and mechanical stimulation. *Stem Cell Research and Therapy*.

[13] Ye K., Traianedes K., Robins S. A., Choong P. F. M., Myers D. E. (2018). Osteochondral repair using an acellular dermal matrix-pilot in vivo study in a rabbit osteochondral defect model. *Journal of Orthopaedic Research*.

[14] Veronesi F., Cadossi M., Giavaresi G (2015). Pulsed electromagnetic fields combined with a collagenous scaffold and bone marrow concentrate enhance osteochondral regeneration: an in vivo study Orthopedics and biomechanics. *BMC Musculoskeletal Disorders*.

[15] Schmal H., Kowal J., Kassem M. (2018). Comparison of regenerative tissue quality following matrix-associated cell implantation using amplified chondrocytes compared to synovium-derived stem cells in a rabbit model for cartilage lesions. *Stem Cells Int*.

[16] Wong C.-C., Chen C.-H., Chiu L.-H. (2018). Facilitating in vivo articular cartilage repair by tissue-engineered cartilage grafts produced from auricular chondrocytes. *The American Journal of Sports Medicine*.

[17] Fekrazad R., Eslaminejad M. B., Shayan A. M. (2016). Effects of photobiomodulation and mesenchymal stem cells on articular cartilage defects in a rabbit model. *Photomedicine and Laser Surgery*.

[18] Kazemnejad S., Khanmohammadi M., Mobini S. (2016). Comparative repair capacity of knee osteochondral defects using regenerated silk fiber scaffolds and fibrin glue with/without autologous chondrocytes during 36 weeks in rabbit model. *Cell and Tissue Research*.

[19] Betz V. M., Keller A., Foehr P. (2017). BMP-2 gene activated muscle tissue fragments for osteochondral defect regeneration in the rabbit knee. *The Journal of Gene Medicine*.

[20] Shimomura K., Moriguchi Y., Ando W. (2014). Osteochondral repair using a scaffold-free tissue-engineered construct derived from synovial mesenchymal stem cells and a hydroxyapatite-based artificial bone. *Tissue Engineering Part A*.

[21] Chung E. J., Kodali P., Laskin W., Koh J. L., Ameer G. A. (2011). Long-term in vivo response to citric acid-based nanocomposites for orthopaedic tissue engineering. *Journal of Materials Science: Materials in Medicine*.

[22] Guzmán-Morales J., Lafantaisie-Favreau C.-H., Chen G., Hoemann C. D. (2014). Subchondral chitosan/blood implant-guided bone plate resorption and woven bone repair is coupled to hyaline cartilage regeneration from microdrill holes in aged rabbit knees. *Osteoarthritis and Cartilage*.

[23] Lafantaisie-Favreau C. H., Guzmán-Morales J., Sun J. (2013). Subchondral pre-solidified chitosan/blood implants elicit reproducible early osteochondral wound-repair responses including neutrophil and stromal cell chemotaxis, bone resorption and repair, enhanced repair tissue integration and delayed matrix deposition. *BMC Musculoskeletal Disorders*.

[24] Cheuk Y.-C., Wong M. W.-N., Lee K.-M., Fu S.-C. (2011). Use of allogeneic scaffold-free chondrocyte pellet in repair of osteochondral defect in a rabbit model. *Journal of Orthopaedic Research*.

[25] Vaseenon T., Tochigi Y., Heiner A. D. (2011). Organ-level histological and biomechanical responses from localized osteoarticular injury in the rabbit knee. *Journal of Orthopaedic Research*.

[26] Zhang S., Chen L., Jiang Y. (2013). Bi-layer collagen/microporous electrospun nanofiber scaffold improves the osteochondral regeneration. *Acta Biomaterialia*.

[27] Jansen E. J. P., Pieper J., Gijbels M. J. J. (2009). PEOT/PBT based scaffolds with low mechanical properties improve cartilage repair tissue formation in osteochondral defects. *Journal of Biomedical Materials Research Part A*.

[28] Bayat M., Javadieh F., Dadpay M. (2009). Effect of He-Ne laser radiation on healing of osteochondral defect in rabbit: a histological study. *The Journal of Rehabilitation Research and Development*.

[29] Filardo G., Perdisa F., Gelinsky M. (2018). Novel alginate biphasic scaffold for osteochondral regeneration: an in vivo evaluation in rabbit and sheep models. *Journal of Materials Science: Materials in Medicine*.

[30] Martin-Hernandez C., Cebamanos-Celma J., Molina-Ros A., Ballester-Jimenez J. J., Ballester-Soleda J. (2010). Regenerated cartilage produced by autogenous periosteal grafts: a histologic and mechanical study in rabbits under the influence of continuous passive motion. *Arthroscopy: The Journal of Arthroscopic and Related Surgery*.

[31] Javadieh F., Bayat M., Torkaman G. (2010). Evaluation of low-level laser therapy with a He-Ne laser on the healing of an osteochondral defect using a biomechanical test. *Photomedicine and Laser Surgery*.

[32] Oncan T., Demirağ B., Ermutlu C., Yalçinkaya U., Özkan L. (2013). Effect of low-dose irradiation on structural and mechanical properties of hyaline cartilage-like fibrocartilage. *Acta Orthopaedica et Traumatologica Turcica*.

[33] Rahman R., Mohamad Sukri N., Md Nazir N. (2015). Evaluation of three dimensional construct engineered from poly(Lactic-co-glycolic acid)/fibrin hybrid scaffold using rabbit bone marrow mesenchymal stem cells for osteochondral defect repair. *Jurnal Teknologi*.

[34] Shimomura K., Moriguchi Y., Nansai R. (2017). Comparison of 2 different formulations of artificial bone for a hybrid implant with a tissue-engineered construct derived from synovial mesenchymal stem cells: a study using a rabbit osteochondral defect model. *The American Journal of Sports Medicine*.

[35] Yin H., Wang Y., Sun X. (2018). Functional tissue-engineered microtissue derived from cartilage extracellular matrix for articular cartilage regeneration. *Acta Biomaterialia*.

[36] Wang C. C., Yang K. C., Lin K. H. (2016). Expandable scaffold improves integration of tissue-engineered cartilage: an in vivo study in a rabbit model. *Tissue Engineering Part A*.

[37] Jia S., Zhang T., Xiong Z., Pan W., Liu J., Sun W. (2015). In vivo evaluation of a novel oriented scaffold-BMSC construct for enhancing full-thickness articular cartilage repair in a rabbit model. *PLoS One*.

[38] Wada S., Kitamura N., Nonoyama T. (2016). Hydroxyapatite-coated double network hydrogel directly bondable to the bone: biological and biomechanical evaluations of the bonding property in an osteochondral defect. *Acta Biomaterialia*.

[39] Mredha M. T. I., Kitamura N., Nonoyama T. (2017). Anisotropic tough double network hydrogel from fish collagen and its spontaneous in vivo bonding to bone. *Biomaterials*.

[40] Wang Q., Zhang H., Gan H., Wang H., Li Q., Wang Z. (2018). Application of combined porous tantalum scaffolds loaded with bone morphogenetic protein 7 to repair of osteochondral defect in rabbits ^∗^. *International Orthopaedics*.

[41] Xiangyu L., Pingguo D., Jingming G. (2018). Bilayered PLGA/PLGA-HAp composite scaffold for osteochondral tissue engineering and tissue regeneration. *ACS Biomaterials Science and Engineering*.

[42] Cao F., Qi J., Song H. (2018). Tsmu solution improves rabbit osteochondral allograft preservation and transplantation outcome. *Cell and Tissue Banking*.

[43] Qiang Y., Yanhong Z., Jiang P. (2014). Xenoimplantation of an extracellular-matrix-derived, biphasic, cell-scaffold construct for repairing a large femoral-head high-load-bearing osteochondral defect in a canine model. *The ScientificWorld Journal*.

[44] McCarty E. C., Fader R. R., Mitchell J. J., Glenn R. E., Potter H. G., Spindler K. P. (2016). Fresh osteochondral allograft versus autograft. *The American Journal of Sports Medicine*.

[45] Baba R., Onodera T., Matsuoka M. (2018). Bone marrow stimulation technique augmented by an ultrapurified alginate gel enhances cartilage repair in a canine model. *The American Journal of Sports Medicine*.

[46] Yang Q., Peng J., Lu S. B. (2011). Evaluation of an extracellular matrix-derived acellular biphasic scaffold/cell construct in the repair of a large articular high-load-bearing osteochondral defect in a canine model. *Chinese Medical Journal*.

[47] Cook J. L., Stannard J. P., Stoker A. M. (2016). Importance of donor chondrocyte viability for osteochondral allografts. *The American Journal of Sports Medicine*.

[48] Cook J. L., Stoker A. M., Stannard J. P. (2014). A novel system improves preservation of osteochondral allografts. *Clinical Orthopaedics and Related Research*.

[49] He A., Liu L., Luo X. (2017). Repair of osteochondral defects with in vitro engineered cartilage based on autologous bone marrow stromal cells in a swine model. *Scientific Reports*.

[50] Ho S. T. B., Hutmacher D. W., Ekaputra A. K., Hitendra D., Hui J. H. (2010). The evaluation of a biphasic osteochondral implant coupled with an electrospun membrane in a large animal model. *Tissue Engineering Part A*.

[51] Christensen B. B., Foldager C. B., Olesen M. L., Hede K. C., Lind M. (2016). Implantation of autologous cartilage chips improves cartilage repair tissue quality in osteochondral defects. *The American Journal of Sports Medicine*.

[52] Jung M., Kaszap B., Redöhl A. (2009). Enhanced early tissue regeneration after matrix-assisted autologous mesenchymal stem cell transplantation in full thickness chondral defects in a minipig model. *Cell Transplantation*.

[53] Gotterbarm T., Breusch S. J., Vilei S. B., Mainil-Varlet P., Richter W., Jung M. (2013). No effect of subperiosteal growth factor application on periosteal neo-chondrogenesis in osteoperiosteal bone grafts for osteochondral defect repair. *International Orthopaedics*.

[54] Zuo Q., Cui W., Liu F., Wang Q., Chen Z., Fan W. (2016). Utilizing tissue-engineered cartilage or BMNC-PLGA composites to fill empty spaces during autologous osteochondral mosaicplasty in porcine knees. *Journal of Tissue Engineering and Regenerative Medicine*.

[55] Menendez M. I., Clark D. J., Carlton M. (2011). Direct delayed human adenoviral BMP-2 or BMP-6 gene therapy for bone and cartilage regeneration in a pony osteochondral model. *Osteoarthritis and Cartilage*.

[56] van Bergen C. J. A., Kerkhoffs G. M. M. J., Özdemir M. (2013). Demineralized bone matrix and platelet-rich plasma do not improve healing of osteochondral defects of the talus: an experimental goat study. *Osteoarthritis and Cartilage*.

[57] Jurgens W. J. F. M., Kroeze R. J., Zandieh-Doulabi B. (2013). One-step surgical procedure for the treatment of osteochondral defects with adipose-derived stem cells in a caprine knee defect: a pilot study. *BioResearch Open Access*.

[58] Cunniffe G. M., Díaz-Payno P. J., Sheehy E. J. (2019). Tissue-specific extracellular matrix scaffolds for the regeneration of spatially complex musculoskeletal tissues. *Biomaterials*.

[59] Marquass B., Somerson J. S., Hepp P. (2010). A novel MSC-seeded triphasic construct for the repair of osteochondral defects. *Journal of Orthopaedic Research*.

[60] Bernstein A., Niemeyer P., Salzmann G. (2013). Microporous calcium phosphate ceramics as tissue engineering scaffolds for the repair of osteochondral defects: histological results. *Acta Biomaterialia*.

[61] Bell A. D., Lascau-Coman V., Sun J. (2013). Bone-induced chondroinduction in sheep Jamshidi biopsy defects with and without treatment by subchondral chitosan-blood implant. *Cartilage*.

[62] Jia S., Wang J., Zhang T. (2018). Multilayered scaffold with a compact interfacial layer enhances osteochondral defect repair. *ACS Applied Materials and Interfaces*.

[63] Kon E., Filardo G., Robinson D. (2014). Osteochondral regeneration using a novel aragonite-hyaluronate bi-phasic scaffold in a goat model. *Knee Surgery, Sports Traumatology, Arthroscopy*.

[64] Kon E., Filardo G., Delcogliano M. (2010). Platelet autologous growth factors decrease the osteochondral regeneration capability of a collagen-hydroxyapatite scaffold in a sheep model. *BMC Musculoskeletal Disorders*.

[65] Manunta A. F., Zedde P., Pilicchi S. (2016). The use of embryonic cells in the treatment of osteochondral defects of the knee: an ovine in vivo study. *Joints*.

[66] Nosewicz T. L., Reilingh M. L., Wolny M., van Dijk C. N., Duda G. N., Schell H. (2014). Influence of basal support and early loading on bone cartilage healing in press-fitted osteochondral autografts. *Knee Surgery, Sports Traumatology, Arthroscopy*.

[67] Schleicher I., Lips K. S., Sommer U. (2013). Biphasic scaffolds for repair of deep osteochondral defects in a sheep model. *Journal of Surgical Research*.

[68] Mayr H. O., Klehm J., Schwan S. (2013). Microporous calcium phosphate ceramics as tissue engineering scaffolds for the repair of osteochondral defects: biomechanical results. *Acta Biomaterialia*.

[69] Maia F. R., Carvalho M. R., Oliveira J. M., Reis R. L. (2018). Tissue engineering strategies for osteochondral repair. *Osteochondral Tissue Engineering*.

[70] da Cunha Cavalcanti F. M. M., Doca D., Cohen M., Ferretti M. (2012). Updating on diagnosis and treatment of chondral lesion of the knee. *Revista Brasileira de Ortopedia (English Edition)*.

[71] Kelly D. J., Prendergast P. J. (2006). Prediction of the optimal mechanical properties for a scaffold used in osteochondral defect repair. *Tissue Engineering*.

[72] Hoemann C., Kandel R., Roberts S. (2011). International cartilage repair society (ICRS) recommended guidelines for histological endpoints for cartilage repair studies in animal models and clinical trials. *Cartilage*.

[73] Rutgers M., van Pelt M. J. P., Dhert W. J. A., Creemers L. B., Saris D. B. F. (2010). Evaluation of histological scoring systems for tissue-engineered, repaired and osteoarthritic cartilage. *Osteoarthritis and Cartilage*.

[74] Orth P., Zurakowski D., Wincheringer D., Madry H. (2012). Reliability, reproducibility, and validation of five major histological scoring systems for experimental articular cartilage repair in the rabbit model. *Tissue Engineering Part C: Methods*.

[75] Erisken C., Kalyon D. M., Wang H. (2010). Viscoelastic and biomechanical properties of osteochondral tissue constructs generated from graded polycaprolactone and beta-tricalcium phosphate composites. *Journal of Biomechanical Engineering*.

[76] Boi M., Marchiori G., Berni M. (2019). Nanoindentation: an advanced procedure to investigate osteochondral engineered tissues. *Journal of the Mechanical Behavior of Biomedical Materials*.

[77] Chu C. R., Szczodry M., Bruno S. (2010). Animal models for cartilage regeneration and repair. *Tissue Engineering Part B: Reviews*.

[78] Glatt V., Evans C. H., Stoddart M. J. (2019). Regenerative rehabilitation: the role of mechanotransduction in orthopaedic regenerative medicine. *Journal of Orthopaedic Research*.

[79] Fahy N., Alini M., Stoddart M. J. (2018). Mechanical stimulation of mesenchymal stem cells: implications for cartilage tissue engineering. *Journal of Orthopaedic Research*.

[80] Mauck R. L., Soltz M. A., Wang C. C. (2000). Functional tissue engineering of articular cartilage through dynamic loading of chondrocyte-seeded agarose gels. *Journal of Biomechanical Engineering*.

[81] Ng K. W., Mauck R. L., Wang C. C. (2009). Duty cycle of deformational loading influences the growth of engineered articular cartilage. *Cellular and Molecular Bioengineering*.

[82] Dossumbekova A., Anghelina M., Madhavan S. (2007). Biomechanical signals inhibit IKK activity to attenuate NF-κB transcription activity in inflamed chondrocytes. *Arthritis and Rheumatology*.

